# 3D Printing of Anisotropic Hydrogels with Bioinspired Motion

**DOI:** 10.1002/advs.201800703

**Published:** 2018-11-22

**Authors:** Hakan Arslan, Amirali Nojoomi, Junha Jeon, Kyungsuk Yum

**Affiliations:** ^1^ Department of Materials Science and Engineering University of Texas at Arlington Arlington TX 76019 USA; ^2^ Department of Mechanical and Aerospace Engineering University of Texas at Arlington Arlington TX 76019 USA; ^3^ Department of Chemistry and Biochemistry University of Texas at Arlington Arlington TX 76019 USA

**Keywords:** 3D printing, actuators, anisotropic hydrogels, bioinspired design, shape morphing

## Abstract

Motion in biological organisms often relies on the functional arrangement of anisotropic tissues that linearly expand and contract in response to external signals. However, a general approach that can implement such anisotropic behavior into synthetic soft materials and thereby produce complex motions seen in biological organisms remains a challenge. Here, a bioinspired approach is presented that uses temperature‐responsive linear hydrogel actuators, analogous to biological linear contractile elements, as building blocks to create three‐dimensional (3D) structures with programmed motions. This approach relies on a generalizable 3D printing method for building 3D structures of hydrogels using a fugitive carrier with shear‐thinning properties. This study demonstrates that the metric incompatibility of an orthogonally growing bilayer structure induces a saddle‐like shape change, which can be further exploited to produce various bioinspired motions from bending to twisting. The orthogonally growing bilayer structure undergoes a transition from a stretching‐dominated motion to a bending‐dominated motion during its shape transformation. The modular nature of this approach, together with the flexibility of additive manufacturing, enables the fabrication of multimodular 3D structures with complex motions through the assembly of multiple functional components, which in turn consist of simple linear contractile elements.

Nature has evolved a variety of soft materials that change their shapes and properties in response to internal signals or environmental cues.^1^, ^2^, ^3^, ^4^, ^5^, ^6^ Inspired by such materials, researchers have developed stimuli‐responsive, self‐shaping materials using shape‐memory polymers,^7^, ^8^ liquid crystalline polymers,^9^, ^10^ and hydrogels.^11^, ^12^, ^13^, ^14^, ^15^ Among these materials, hydrogels are promising for bioinspired and biomedical applications, such as soft robotics, artificial muscles, and smart medicine, owing to their physical properties similar to biological soft tissues.^11^, ^12^, ^13^, ^14^, ^15^, ^16^, ^17^, ^18^ Such biomimetic properties include soft polymer structures with high water content, biocompatibility, and reversible volume changes in response to external stimuli, such as temperature, light, pH, chemicals, and biomolecules.^13^, ^14^, ^15^, ^16^, ^17^, ^18^ However, most synthetic hydrogels are structurally isotropic and thus show isotropic material properties and actuation.^18^ In contrast, biological tissues adopt anisotropic structures with hierarchical architectures and thus display anisotropic properties and actuation in their fundamental units.^18^ For example, muscle tissues use anisotropic microstructures, from actin and myosin in sarcomeres to muscle fibers, to generate macroscopic linear contractions.^18^, ^19^, ^20^ Plants produce motion through the anisotropic swelling and shrinkage of sclerenchyma tissues in response to environmental stimuli, such as humidity, touch, and light.^2^, ^3^, ^13^, ^21^, ^22^, ^23^

The anisotropy plays a critical role in shape changes and movements of biological organisms.^2^, ^3^, ^13^, ^18^, ^19^, ^20^, ^21^, ^22^, ^23^, ^24^ They often achieve complex motions through the spatial arrangement of simple linear contractile elements, rather than relying on specifically designed actuators with complex structures (often used in man‐made machines).^6^, ^19^, ^20^ For example, the heart generates twisting and compressive motions through the helical and circumferential arrangement of the outer two muscle layers.^19^, ^20^ Plants have evolved mechanisms that convert the anisotropic swelling and shrinking of tissues into various motions, including bending, coiling, and twisting.^2^, ^3^, ^13^, ^18^, ^19^, ^20^, ^21^, ^22^, ^23^, ^24^ Inspired by such mechanisms, researchers have prepared linearly‐actuating anisotropic hydrogels and their bilayer structures to create various bioinspired 3D structures.^13^, ^18^, ^23^ The linear anisotropic behaviors have been achieved, for example, by controlling the orientation of stiff reinforcing elements in a hydrogel matrix using magnetic fields^23^ and flow‐induced shear forces during 3D printing.^13^ However, these approaches need to couple fabrication processes (e.g., crosslinking of hydrogels and printing of filaments) with reinforcement alignment, limiting achievable 3D architectures. For example, the approach using magnetic fields involves multiple steps, such as rotating a magnetic field, addition of a precursor solution for each layer, and manual cutting.^23^ In addition, although they have demonstrated reversible shape changes, previous studies have mainly focused on the hydration‐driven formation of 3D shapes (rather than their stimuli‐responsive motions).^13^, ^23^ A strategy that can directly implement linear contractile elements into 3D architectures could provide a simple yet versatile way to program various motions into 3D structures. 3D printing has great potential in this regard, but printing self‐supporting 3D structures of hydrogels, including those of stimuli‐responsive hydrogels, has not been fully achieved.^5^, ^13^, ^25^

Here, we present a bioinspired approach that uses linear hydrogel actuators, analogous to biological linear contractile elements, as building blocks to create temperature‐responsive 3D structures with programmed motions (**Figure** **1**a). This approach relies on a generalizable method for printing 3D structures of hydrogels from low viscosity precursor solutions using a gel‐phase fugitive carrier with shear‐thinning properties (Figure 1b–d; Figure S1, Supporting Information). Building on this capability and inspired by biological anisotropic tissues, we printed linear hydrogel actuators (or linear contractile elements) composed of temperature‐unresponsive poly(ethylene glycol) (PEG) reinforcement elements in a temperature‐responsive poly(*N*‐isopropylacrylamide) (PNIPAM) matrix (Figure 1a). The anisotropic PEG pattern in the isotropic PNIPAM matrix restricts the temperature‐responsive swelling and shrinkage of the matrix along the direction of continuous PEG filaments (reinforcement direction). This design thereby induces anisotropic actuation perpendicular to the reinforcement direction. We demonstrate that the metric incompatibility of a bilayer structure that consists of orthogonally oriented linear contractile elements (orthogonally growing bilayer structure) induces a saddle‐like shape change, the ubiquitous mode of the bilayer structure (Figure 1a).^3^, ^26^ This saddle‐like shape change (metric incompatibility) can be further exploited to produce various motions from bending to twisting by controlling the geometry and orientation of the elements (Figure 1a; Figure S2, Supporting Information).^3^, ^26^ Increasing the aspect ratio of the bilayer structure induces a pure bending‐like motion (Figure 1a; Figure S2, Supporting Information).^3^, ^26^ Controlling the angle θ between the long axis of the structure and the direction of intrinsic curvature produces bending, coiling, and twisting motions with θ of 0°, 22.5°, and 45°, respectively (Figure 1a; Figure S2, Supporting Information).^3^, ^26^ Our results reveal that the orthogonally growing bilayer structure undergoes a transition from a stretching‐dominated motion to a bending‐dominated motion during its shape transformation from the swelled state to the shrunk state. The modular nature of our approach, in combination with our flexible 3D printing method, enables programming of complex motions into 3D structures through the assembly of multiple functional components, which in turn consist of simple linear contractile elements.

**Figure 1:**
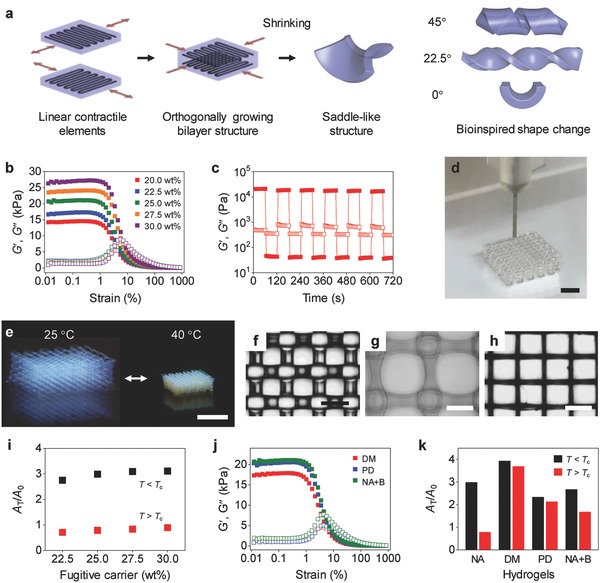
3D printing of orthogonally growing bilayer structures of hydrogels with programmed motions. a) Schematic illustrating the 3D printing‐based process to create 3D structures with programmed motions using a bilayer structure of orthogonally oriented linear contractile elements. The arrows indicate the direction of anisotropic actuation, perpendicular to the PEG reinforcement direction. The angles θ shown in the legend (right figures) indicate the angle between the long axis of a bilayer structure and the direction of intrinsic curvature (Figure S2, Supporting Information). b) *G*′ and *G*″ of PNIPAM inks (10 wt% NIPAM and 1 wt% PEGDA) with different concentrations of the fugitive carrier (20–30 wt% as shown in the legend) on oscillatory strain sweeps (0.01%–1000%) at a frequency of 1 Hz. c) Step–strain measurement of a PNIPAM ink (10 wt% NIPAM, 1 wt% PEGDA, and 25 wt% fugitive carrier) with oscillatory strain steps between 0.5% and 250% at a frequency of 10 Hz. d) 3D printing of a multilayer lattice structure using a 200 µm nozzle. e) Temperature‐responsive reversible volume change of a PNIPAM structure. f–h) Optical microscope images (top view) of an as‐printed 3D structure (f) and the structure at the swelled state (25 °C) (g) and the shrunk state (40 °C) (h). i) Areal swelling (black) and shrinking (red) ratios (*A*_T_/*A*_0_) of 3D structures of PNIPAM printed with different concentrations of the fugitive carrier. *A*_T_ and *A*_0_ are the areas of the top surface of the structures at temperature *T* and as‐printed structures, respectively. *T*_c_ is the volume phase transition temperature of PNIPAM (≈32.5 °C). j) *G*′ and *G*″ of DM (10 wt% DMA and 1 wt% PEGDA), PD (11 wt% PEGDA), and NA+B (10 wt% NIPAM and 1 wt% BIS) inks on oscillatory strain sweeps (0.01%–1000%) at a frequency of 1 Hz. k) Areal swelling (black) and shrinking (red) ratios of the inks. NA represents the PNIPAM ink (10 wt% NIPAM and 1 wt% PEGDA). Scale bars, 2 mm (d); 5 mm (e); 500 µm (f–h).

Our 3D printing approach relies on a gel‐phase ink with shear‐thinning properties (Figure 1b,c). The ink consists of a hydrogel precursor solution and a gel‐phase fugitive carrier. We used a triblock copolymer (polyethylene oxide (PEO)–polypropylene oxide (PPO)–PEO or Pluronic F127) as our carrier because of its excellent 3D printability with shear‐thinning and thermally reversible gelation behaviors (Figures S3 and S4, Supporting Information).^27^, ^28^ The shear‐thinning behavior of the carrier renders our ink 3D printable, whereas the thermally reversible gel‐to‐fluid transition allows for the complete removal of the carrier from printed structures. In addition, the printed structures of the carrier function as a template to form the primary hydrogel through photopolymerization and crosslinking after printing. 3D printability of our inks does not rely on the material properties of hydrogel precursors, but those of the fugitive carrier. This approach can thus be generally applicable to various hydrogel systems, regardless of their rheological properties before printing (i.e., those of hydrogel precursors) and the material properties of the final printed hydrogels (i.e., crosslinked hydrogels). In contrast, as hydrogel precursors with low viscosity are not 3D printable, most previous methods have modified the rheological properties of printing inks to achieve 3D printability (e.g., viscosity modulation, pre‐crosslinking, addition of nanoparticles, and guest–host shear‐thinning formulation).^13^, ^25^, ^29^, ^30^, ^31^ Such modifications often couple with and thus alter the properties of the final printed hydrogels or work only for specific hydrogel formulations, limiting the palette of 3D printable hydrogels.^25^

To demonstrate our printing approach, we first investigated the rheological properties of our inks (Figure 1b,c). We designed gel‐phase inks (10 wt% *N*‐isopropylacrylamide (NIPAM) and 1 wt% poly(ethylene glycol) diacrylate (PEGDA)) with the fugitive carrier (20.0–30.0 wt% slightly below and above its critical micelle concentration of ≈21 wt%) for printing PNIPAM hydrogels.^27^, ^28^ We selected PNIPAM as our model system because of its temperature‐responsive volume change useful for building soft actuators.^32^ The rheological measurements indicate that our PNIPAM inks are in gel phase (shear storage modulus *G*′ > shear loss modulus *G*″) and have excellent shear‐thinning and recovery properties, critical for 3D printing (Figure 1b,c). The *G*′ value rapidly decreases above critical strain (≈1%) and becomes lower than *G*″ (*G*′–*G*″ crossover) above crossover strain of 4% to 8%, reflecting yielding of the inks and their transition to a liquid‐like state (*G*′ < *G*″), required for extruding the inks (Figure 1b). The step–strain measurements show the fast recovery of the ink from a liquid‐like state (*G*′ < *G*″) to a solid‐like state (*G*′ > *G*″) upon reduction of applied strain following shear‐thinning at high strain (Figure 1c). The rapid recovery is important for high‐resolution 3D printing, as it prevents the flow of the ink after extrusion (often observed in liquid‐phase inks).^25^ In addition, the *G*′ value of our inks is high enough to support as‐printed structures before crosslinking (*G*′ > ≈15 kPa at low strain).

To demonstrate 3D printability of our inks, we printed multilayer lattice structures using a 200 µm nozzle (Figure 1d; Figure S1, Supporting Information). We define the inks that produce well‐defined, self‐supporting multilayer structures as 3D printable. Our PNIPAM inks with the fugitive carrier (>22.5 wt%) show excellent 3D printability (Figure 1d; Figure S5, Supporting Information). After printing, we formed PNIPAM networks within the printed structures through photopolymerization and crosslinking using the printed filaments of the carrier as a template. We then removed the carrier by immersing the structures in water at 4 °C. The resulting 3D structures, thus composed of pure PNIPAM hydrogels, exhibit the characteristic volume change behavior while maintaining their lattice structures (Figure 1e–h). The concentration of the carrier does not affect the behavior (Figure 1i). The precursor solutions without the carrier are low viscosity liquids and thus not 3D printable.

Our results suggest that the shear‐thinning and rapid recovery behavior of our inks with the fugitive carrier (>22.5 wt%) enables high‐resolution 3D printing of hydrogels (Figure 1f–h). These properties allow the inks to be extruded through a 200 µm nozzle while maintaining its filamentary structure after extrusion. In contrast, the ink (10 wt% NIPAM and 1 wt% PEGDA) with the fugitive carrier (20 wt%) below its critical micelle concentration (≈21 wt%) shows a shear‐thinning behavior (Figure 1b), but it yields deformed 3D structures with irregular patterns and circular spaces between filaments, reflecting spreading of the ink and the formation of menisci after extrusion (Figure S6, Supporting Information). The deformation of as‐printed structures indicates that *G*′ of ≈14 kPa may not be sufficient to sustain the weight of printed filaments before crosslinking, suggesting a critical modulus (*G*′ of ≈15 kPa) required for printing self‐supporting 3D structures (Figure 1b).^27^

A key advantage of our printing approach is that it is generalizable to photocrosslinkable hydrogel systems, regardless of their physical properties before and after printing. To demonstrate this capability, we printed hydrogel 3D structures using gel‐phase inks that consist of the fugitive carrier (25.0 wt%) and various precursor solutions: (i) 10 wt% *N*,*N*‐dimethylacrylamide (DMA) and 1 wt% PEGDA (DM), (ii) 11 wt% PEGDA (PD), and (iii) 10 wt% NIPAM and 1 wt% *N*,*N*′‐methylene bisacrylamide (BIS) (NA+B). Strain‐dependent oscillatory rheology of the inks shows their shear‐thinning properties (Figure 1j). As expected from their rheological properties, all inks are 3D printable, illustrating the versatility of our printing method (Figure S7, Supporting Information). The printed structures exhibit the characteristic swelling and shrinking behaviors of the primary hydrogels (Figure 1k).

Taking advantage of our 3D printing method, we created 3D structures with anisotropic actuation (linear contractile elements) using two types of hydrogels (**Figure** **2**). Inspired by biological anisotropic tissues, we printed anisotropic PEG patterns in isotropic PNIPAM matrices, in which the PEG patterns work as artificial reinforcing elements (Figure 2a–d). The PEG patterns were printed in the middle layer of the structures to prevent bending and achieve pure stretching motions. The basic concept is to restrict the swelling and shrinking of PNIPAM structures in the reinforcement direction, as observed in anisotropic biological tissues (e.g., those of pine cones, wheat awns, and *Bauhinia* seed pods reinforced with cellulose microfibrils).^3^, ^21^, ^22^, ^23^ As designed, the 3D structures with anisotropic PEG patterns linearly actuate by ≈210% in the direction perpendicular to the PEG reinforcement (Figure 2a–d). The actuation in the longitudinal direction (≈110% strain), the major actuation direction, from a shrunk state to a swelled state is around sixfold higher than the actuation in the transverse direction (≈20% strain). The direction of actuation can be controlled by the orientation of PEG patterns (Figure 2a–d). In addition to anisotropic actuation, the 3D structures show a high rate of actuation (≈75% min^−1^ in the linear range) compared to conventional 3D hydrogel structures (Figure 2e; Figure S8, Supporting Information). This rate is much higher than that of homogeneous PNIPAM hydrogels (e.g., ≈60 min required for 15% volume shrinkage for 2 mm‐thick disks)^33^ and comparable to those of fast‐responsive hydrogels, such as nanostructured hydrogels (>2 min required for 50% volume shrinkage for 10 mm‐thick disks)^34^ and nanocomposite cryogels (typically >5 min for 50% volume shrinkage for cylindrical hydrogels with diameter of 6 mm).^35^ The interfilament microchannels facilitate the transport of water through 3D structures, leading to fast actuation.

**Figure 2:**
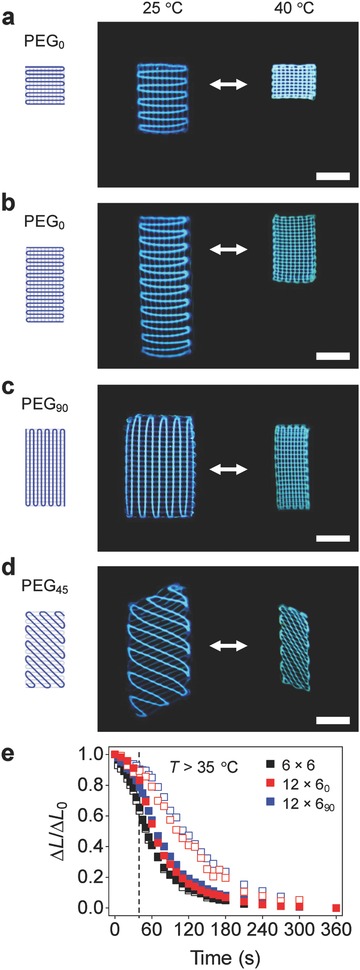
3D structures of hydrogels with anisotropic actuation. a) Hydrogel linear actuator with an as‐printed length and width of 6 mm. b–d) Hydrogel linear actuators with an as‐printed length and width of 12 and 6 mm, respectively, that actuate in the direction at 0° (b), 90° (c), and 45° (d) with respect to the long axis. The structures (a–d) consist of two layers of PNIPAM hydrogels, one layer of PEG pattern, and two layers of PNIPAM hydrogels from the bottom layer to the top layer and have an as‐printed thickness of 1 mm. The figures show the schematics of PEG patterns (dark blue lines) in PNIPAM hydrogels of as‐printed structures (left), the PNIPAM structures with PEG patterns (shown in dark blue lines) at the swelled state (middle), and the structures at the shrunk state (right). e) Changes in the relative length Δ*L*/Δ*L*_0_ of the structure shown in Figure 1e (black squares; as‐printed thickness of 2 mm) and the linear actuators shown in (b) (red squares) and (c) (blue squares) upon rapid increase in temperature from 24 to 50 °C. Δ*L*/Δ*L*_0_ = (*L* − *L*_50_)/(*L*_24_ − *L*_50_), where *L*, *L*_50_, and *L*_24_ are the lengths of the structures at the time of measurement, at the shrunk state (*T* = 50 °C), and at the swelled state (*T* = 24 °C), respectively. The dashed line indicates the time when the solution temperature reaches ≈35 °C (Figure S8, Supporting Information). The closed and open squares represent the relative lengths along the major actuation and transverse directions, respectively. Scale bars, 5 mm.

We next explored whether we can design 3D structures with programmed motions through the modular assembly of linear contractile elements (**Figure** **3**). This strategy takes the inspiration from plant organs that achieve various movements using a bilayer structure of orthogonally oriented tissues with uniaxial swelling and shrinking behaviors. We thus considered such an orthotropically growing bilayer structure with a square shape (Figure 3a). The structure was constructed by printing a linear contractile element (e.g., one shown in Figure 2a) on top of another element in the perpendicular direction. The structure reversibly transforms its configuration from a near‐planar shape at the swelled state to a saddle‐like shape with two principal curvatures with opposite signs (Gaussian curvature *K* = *k*_1_ *k*_2_ < 0, where *k*_1_ and *k*_2_ are principal curvatures) at the shrunk state (Figure 3a).

**Figure 3:**
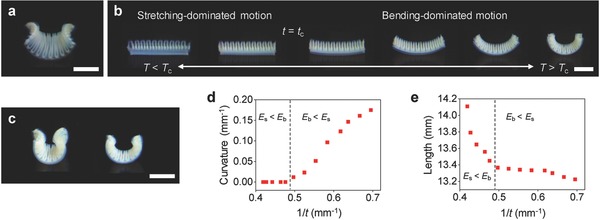
Orthogonally growing bilayer structures with saddle‐like shape change and bending motion. a) Saddle‐like shape of an orthogonally growing bilayer structure (as‐printed size: 12 mm × 12 mm) at the shrunk state. b) Bending motion of an orthogonally growing bilayer structure with a high aspect ratio (as‐printed size: 12 mm × 4.2 mm) along the long axis upon temperature increase from 25 to 40 °C. c) Bending of orthogonally growing bilayer structures with a low aspect ratio (left; as‐printed size: 12 mm × 7.8 mm) and a high aspect ratio (right; as‐printed size: 12 mm × 4.2 mm) at the shrunk state. The structures in (a), (b), and (c) have an as‐printed thickness of ≈1.6 mm. d,e) Curvature (d) and length (e) of the structure shown in (b) along the long axis as a function of 1/*t*. Scale bars, 5 mm.

To understand the shape change, we theoretically considered such an orthogonally growing bilayer structure. The theoretical model based on the elastic energy equivalence between an orthogonally growing bilayer structure and a curved monolayer structure, energetically equivalent to the bilayer structure, provides the relationship between them as follows:

(1) $$\begin{array}{c} a_{r}=\frac{1}{2}\;\left(a_{1}+a_{2}\right),\;\;b_{r}=\frac{3}{4t}\;\left(a_{1}-a_{2}\right) \end{array}$$

where *a*_r_ and *b*_r_ are the first and second fundamental form (reference metrics) of the equivalent monolayer structure in a strain‐free configuration, *a*_1_ and *a*_2_ are the first fundamental forms of the individual layers (linear contractile elements) of the bilayer structure, and *t* is the thickness of the structures.^5^ The energetically equivalent monolayer structure represents the midsurface of the bilayer structure. In general, the actual configuration of the bilayer structure is not strain free and thus the metrics of its midsurface *a*_c_ and *b*_c_ are close to but can be different from *a*_r_ and *b*_r_ (*a*_c_ ≠ *a*_r_ or *b*_c_ ≠ *b*_r_).^3^, ^5^ The structure adopts a residually strained configuration of minimum energy determined by a competition between stretching and bending energies.^3^, ^5^ The orthogonally oriented linear contractile elements have the metrics in the form

(2) $$\begin{array}{c} a_{1}=\left(\begin{array}{cc} \beta^{2} & 0\\ 0 & \alpha^{2} \end{array}\right),\;\;a_{2}=\left(\begin{array}{cc} \alpha^{2} & 0\\ 0 & \beta^{2} \end{array}\right) \end{array}$$

where α and β are the linear growth (swelling or shrinking) factors in the longitudinal (major actuation) and transverse directions, respectively. The metrics of the strain‐free monolayer structure, energetically equivalent to the orthogonally growing bilayer structure, can thus be obtained from Equations (1) and (2).

(3) $$\begin{array}{c} a_{r}=\frac{\alpha^{2}+\beta^{2}}{2}\left(\begin{array}{cc} 1 & 0\\ 0 & 1 \end{array}\right),\;\;b_{r}=\frac{3\left(\alpha^{2}-\beta^{2}\right)}{4t}\;\left(\begin{array}{cc} -1 & 0\\ 0 & 1 \end{array}\right) \end{array}$$

These metrics adopt the Gaussian and mean curvatures

(4) $$\begin{array}{c} K=-\frac{9}{4t^{2}}\;{\left(\frac{\alpha^{2}-\beta^{2}}{\alpha^{2}+\beta^{2}}\right)}^{2}=-k_{0}^{2},\;\;H=0 \end{array}$$

where *K* = det(*a*_r_^−1^ *b*_r_), *H* = (1/2) trace(*a*_r_^−1^ *b*_r_), and *k*_0_ is a principal curvature.^36^ The model suggests that an orthogonally growing bilayer structure induces a saddle‐like shape (*K* = *k*_1_ *k*_2_ < 0 and *k*_1_ = *k*_0_ ≈ −*k*_2_) for any anisotropic growth factors (α ≠ β), as observed in the experimentally created structure (Figure 3a). For typical values of α = 0.85 and β = 1.05 of our linear contractile elements (Figure 2) and *t* = 1.5 mm of a bilayer structure, the model provides *k*_0_ = 0.20 mm^−1^, which agrees well with an experimentally measured *k*_1_ of ≈0.18 mm^−1^ (Figure 3a). The model also predicts *k*_1_ = −*k*_2_ and thus *H* = 0. However, the experimental structure has *k*_1_ > −*k*_2_ ≈ 0.12 mm^−1^. We attribute this behavior to the interaction between the structure and the substrate, which decreases *k*_2_ along the second principal axis.

Inspired by natural hygromorphs,^21^, ^22^ we next converted the saddle‐like shape change into bending motion (Figure 3b). For example, the pine cone scale consists of two tissue layers reinforced perpendicular and parallel to its long axis in a bilayer configuration with a high aspect ratio.^21^, ^22^, ^23^ As the layer reinforced parallel to the long axis restricts its swelling along the long axis and the other layer does not, the reinforcement architecture induces humidity‐driven bending in the opening and closure of the pine cone.^21^, ^22^, ^23^ As observed in the pine cone scale, increasing the aspect ratio of our orthogonally growing bilayer structure along the direction of a principal curvature (θ = 0°) induces bending motion along the long axis (Figure 3b).^21^, ^22^ As the aspect ratio increases, the equilibrium configurations of the bilayer structures at the shrunk state transform from a saddle‐like shape (Figure 3a) to an arc shape (Figure 3b,c; Figure S9, Supporting Information). The structure with a high aspect ratio generates bending motion, macroscopically similar to pure bending along the long axis, while preserving the second curvature (bending) along the short axis.

An important finding is that the bilayer structure undergoes a transition between a stretching‐dominated motion and a bending‐dominated motion during its shape transformation (Figure 3b). Although our linear contractile elements yield significant anisotropic swelling (Figure 2), the bilayer structure forms a near‐planar shape at the swelled state (*T* < *T*_c_), presumably because of the relatively large *t* (Figure 3b). As the stretching and bending energies scale *E*_s_ ∝ *t* and *E*_b_ ∝ *t* ^3^, respectively, it is expected that stretching‐dominated motion is energetically favorable at the swelled state (*E*_s_ < *E*_b_), if *t* is larger than a critical thickness *t*_c_.^11^, ^37^ Our results show that as it shrinks, the bilayer structure indeed undergoes a transition from a stretching‐dominated (linear contractile) motion (*t* > *t*_c_) to a bending‐dominated (pure bending‐like) motion (*t* < *t*_c_) at *t*_c_ ≈ 2.1 mm (Figure 3b; Movie S1, Supporting Information). To further elucidate this mechanism, we monitored the curvature and length of the structure as a function of *t* during its shape transformation (Figure 3d,e). The curvature *k* shows a clear transition from a linear contractile motion (*k* = 0) to a bending motion (*k* ∝ 1/*t*) at *t*_c_ ≈ 2.1 mm (Figure 3d). More interestingly, the rate of length change makes a transition from *dl*/*d*(1/*t*) < 0 to *dl*/*d*(1/*t*) ≈ 0 at *t*_c_ ≈ 2.1 mm. These behaviors indicate that the bending motion is less costly than the linear contractile motion at *t* < *t*_c_. The structure thus tends to preserve its length at *t* < *t*_c_ (Figure 3e). The residual stress developed by the metric incompatibility is therefore mostly relieved by out‐of‐plane bending at *t* < *t*_c_. This mechanism differs from that of bimorph bending (e.g., bimetallic thermostats) with a single curvature induced by the differential growth of the two layers in the same direction (*K* ≥ 0, *H* ≠ 0).^38^ In contrast, the bending motion in this study is driven by the competition between stretching and bending energies (*K* < 0, *H* ≈ 0).

In addition to simple bending, we can exploit the saddle‐like shape change to produce twisting motions (**Figure** **4**a–i). Our inspiration also comes from biological organisms that generate twisting motions through the assembly of linear contractile elements in a bilayer configuration, such as heart tissues^19^, ^20^ and *Bauhinia* seed pods.^3^ For example, the seed pod consists of two orthogonally oriented fibrous tissue layers at 45° with respect to the pod's long axis (θ = 45°).^3^ When drying, the anisotropic shrinkage of the tissue layers in the orthogonal directions leads to the flat‐to‐helical transition, opening the pod.^3^ As observed in the seed pod, an orthogonally growing bilayer structure oriented at 45° with respect to its long axis (θ = 45°) produces twisting motion (Figure 4a). When it shrinks (*T* > *T*_c_), the structure tends to bend along the perpendicular axes (±45° with respect to its longitudinal axis) in two opposite (upward and downward) directions (Figure 4a; Movie S2, Supporting Information). This behavior causes a metric incompatibility, inducing twisting motion.^3^ The metric of such a bilayer structure with θ = 45° can be obtained by rotating the metric for the structure with θ = 0° or 90° in Equation (3) by 45°.

(5) $$b_{r}=\frac{3\left(\alpha^{2}-\beta^{2}\right)}{4t}\;\left(\begin{array}{cc} 0 & 1\\ 1 & 0 \end{array}\right)$$

**Figure 4:**
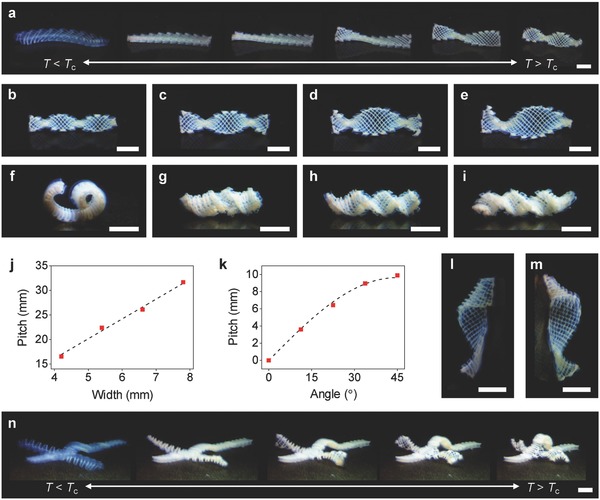
Programming of complex motions. a) Twisting motion of an orthogonally growing structure. b–e) Twisting motions of orthogonally growing structures with an as‐printed length and thickness of 24 and 1.6 mm, respectively, and width of 4.2 mm (b), 5.4 mm (c), 6.6 mm (d), and 7.8 mm (e). The figures show the structures at the shrunk state (*T* > *T*_c_). f–i) Hybrid bending and twisting motions of orthogonally growing bilayer structures (as‐printed size: 24, 4.2, and 1.4 mm in length, width, and thickness, respectively) with θ of 0° (f), 22.5° (g), 33.75° (h), and 45° (i). j) Pitch of the structures shown in (b–e) as a function of width. The dashed line shows a linear fitting (*p* = 4*w*). k) Pitch of the structures shown in (f–i) as a function of θ. The dashed line shows the theoretical prediction based on the seed pod model *p* = (2π/*k*_0_) sin2θ. l,m) Twisting configurations with θ of 22.5° (l) and 112.5° (m), showing reversed handedness. n) Multimodular 3D structure with multiple functional components, showing hybrid motions in response to temperature change from *T* < *T*_c_ (left) to *T* > *T*_c_ (right). Scale bars, 5 mm.

The metric *b*_r_ (curvature tensor) with non‐diagonal matrix elements in Equation (5) reflects pure twisting along the long axis.^3^

We next explored the programmability of twisting motion. In the process of forming a helical twist, the width *w* of the bilayer structure plays a critical role. For example, the theoretical model for the seed pod suggests a transition from the bending‐dominated regime (*w* < *w*_c_, where *w*_c_ is a critical width) to the stretching‐dominated regime (*w* > *w*_c_) with *w*, which yield cylindrical and twisted helices, respectively.^3^ We thus printed bilayer structures with different *w* and characterized their twisting motions by monitoring the pitch *p* and radius *r* of the resulting helical twists at the shrunk state (Figure 4b–e). *p* was found to increase with *w* (*p* ≈ 4*w*), while *r* = 0, allowing us to structurally program twisting motions (Figure 4j).^3^ These results also indicate that the twisting motion of our bilayer structures is in the bending‐dominated regime (*b* ≈ *b*_r_), in which *p* increases with *w*, but *r* = 0.^3^

To further investigate the possibility of programming twisting motions, we studied how the orthogonally growing bilayer structure changes its motion from bending (θ = 0°) to twisting (θ = 45°). Figure 4f–i shows the equilibrium configurations of the structures with the same *w*, but varying θ from 0° to 45° at the shrunk state. As θ increases, the configurations transform from an arc shape (θ = 0°; bending) to a cylindrical helix (θ = 22.5°; hybrid bending and twisting) to a helical twist (θ = 45°; twisting), which mimic various motions of plants, for example, those of pine cone scales,^21^, ^22^ coiled tendrils of climbing plants,^9^, ^24^ and *Bauhinia* seed pods,^3^ respectively. The pitch of the configurations increases with θ, whereas their radius decreases (Figure 4f–i). This behavior agrees well with the theoretical prediction for the seed pod in the bending‐dominated regime *p* = (2π/*k*_0_) sin 2θ (dashed line in Figure 4k),^3^ where the curvature of the arc structure with θ = 0° (Figure 4f) is used as *k*_0_ (*k*_0_ = 0.65 mm^−1^). The structures with θ of 90° to 45° induce the same motions as those with θ of 0° to 45°, whereas those with θ of 90° to 180° produce motions with reversed handedness (Figure 4l,m; Figure S10, Supporting Information). The examples shown in Figures 3 and 4 illustrate a variety of motions achievable by controlling the orientation and geometry of the simple bilayer structure with orthogonally oriented linear contractile elements.

The modular nature of our approach, in combination with the flexibility of our 3D printing method, offers a versatile way to create multimodular 3D structures with complex motions. To demonstrate this capability, we fabricated multimodular 3D structures that consist of multiple functional components (Figure 4n; Movies S3–S5, Supporting Information). The structures show complex motions that combine linear contraction, bending, and twisting. As demonstrated in these examples, the modular approach could potentially enable programming of an unlimited number of motions into 3D structures beyond those of biological counterparts.

Motion in biological systems often relies on a bilayer structure of orthogonally oriented anisotropic tissues that linearly expand and contract. Inspired by the mechanism, we developed an additive manufacturing‐based method that can program complex motions into 3D structures using orthogonally growing bilayer structures. This approach exploits a saddle‐like shape change, the only nontrivial mode of an orthogonally growing bilayer structure,^3^, ^5^ to produce various motions from simple bending to coiling to twisting, mimicking those of biological organisms. The true potential of this approach lies in its ability to achieve complex motions through the modular assembly of functional components with programmed motions. With the design freedom of additive manufacturing, this work opens new ways for not only fabricating soft devices with bioinspired motions, but also studying differential growth‐induced motions in artificial and biological systems.

## Experimental Section

*Preparation of Printing Inks*: The printing inks consisted of a hydrogel precursor solution (11 wt%), a photoinitiator (1 wt% 2,2‐diethoxyacetophenone), and the fugitive carrier (25 wt% Pluronic F127). The printing inks for i) PNIPAM crosslinked with PEGDA, ii) poly(*N*,*N*‐dimethylacrylamide) (PDMA), iii) PEGDA, and iv) PNIPAM crosslinked with BIS were prepared by dissolving i) NIPAM (10 wt%) and PEGDA (1 wt%), ii) DMA (10 wt%) and PEGDA (1 wt%), iii) PEGDA (11 wt%), and iv) NIPAM (10 wt%) and BIS (1 wt%), respectively, with 2,2‐diethoxyacetophenone (1 wt%) and Pluronic F127 (25 wt%) in deionized water at 4 °C. The inks were stirred vigorously for 8 h at 4 °C. The inks were then purged with nitrogen and stored at 4 °C overnight to remove air bubbles before use. Prior to printing, the inks were loaded in 3 mm syringe barrels (Nordson EFD) and stored at room temperature for 2 h. Because they were in a liquid state, the inks at 4 °C could be easily transferred. All chemicals were purchased from Sigma‐Aldrich and used as received.

*Rheological Characterization of Inks*: The rheological properties of the printing inks were measured using a rheometer (DHR‐2, TA Instruments) with a 20 mm‐plate geometry at room temperature. Hydrogel disks of the inks with a diameter of 20 mm were prepared and used at room temperature. To characterize their shear‐thinning properties, *G*′ and *G*′′ of the inks were measured by oscillatory strain sweeps (0.01%–1000%) at a frequency of 1 Hz. To investigate the recovery properties of the inks, step–strain measurements were performed by oscillatory strain steps between 0.5% and 250% at a frequency of 10 Hz.

*3D Printing of Hydrogels*: The 3D printing process is illustrated in Figure S1 in the Supporting Information. Print paths were generated by a G‐code generator (Slic3r) or manually, and simulated and reviewed by a simulation software (CAMotics). 3D structures of hydrogels were printed by an extrusion‐based 3D printer with dual print heads and ultraviolet (UV) light‐emitting diode (LED) curing systems (Inkredible+, Cellink) using 200 µm stainless steel nozzles (Nordson EFD) at room temperature. The 3D structures were designed to have a center‐to‐center distance between filaments of 600 µm and a layer height of 200 µm. The 3D structures were printed at a printing speed of 1–4 mm s^−1^ and printing pressure of 120–280 kPa, depending on *G*′ of printing inks. After printing, the 3D structures were irradiated by UV light (365 nm) for 3 min or longer to polymerize and crosslink the precursor solutions. After forming the primary hydrogels, the fugitive carrier was removed by immersing the crosslinked structures in water at 4 °C for 30 min. To completely remove the fugitive carrier and achieve the equilibrium shapes at the swelled state, the structures were further stored in water at 4 °C for 24 h while exchanging the water every several hours. The temperature‐responsive shape changes of 3D printed structures were characterized in a temperature‐controlled water bath.

## Conflict of Interest

The authors declare no conflict of interest.

## Supporting information

Supplementary

Supplementary

Supplementary

Supplementary

Supplementary

Supplementary
