# Supplementary material for: 3D Printing of Anisotropic Hydrogels with Bioinspired Motion
Source: Adv Sci (Weinh). 2018 Nov 22;6(2):1800703. doi: 10.1002/advs.201800703 (PMC6343088; doi:10.1002/advs.201800703)
Supplement: Supplementary file 1 — Supplementary [file ADVS-6-1800703-s002.pdf]

Copyright WILEY-VCH Verlag GmbH & Co. KGaA, 69469 Weinheim, Germany, 2018.

ADVANCED SCIENCE
Open Access

Supporting Information

for *Adv. Sci.,* DOI: 10.1002/advs.201800703

3D Printing of Anisotropic Hydrogels with Bioinspired Motion

*Hakan Arslan, Amirali Nojoomi, Junha Jeon, and Kyungsuk Yum**

Copyright WILEY-VCH Verlag GmbH & Co. KGaA, 69469 Weinheim, Germany, 2018.

Supporting Information

3D Printing of Anisotropic Hydrogels with Bioinspired Motion

*Hakan Arslan, Amirali Nojoomi, Junha Jeon, and Kyungsuk Yum**

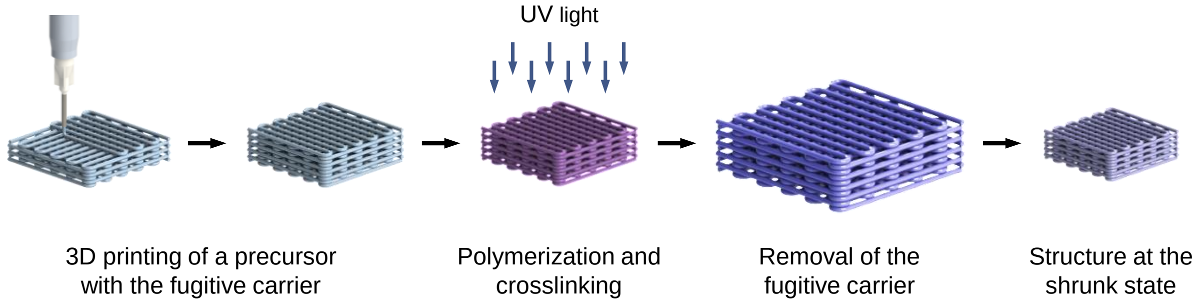

Figure S1. Schematic illustrating 3D printing of hydrogels from low viscosity precursor solutions using a gel-phase fugitive carrier with shear-thinning properties. A 3D structure of a hydrogel is printed with a gel-phase ink (hydrogel precusor with the fugitive carrier). The printed 3D structure is then irradiated by UV light to polymerize and crosslink the hydrogel precursor within the printed structure. After forming the primary hydrogel, the fugitive carrier is removed by immersing the crosslinked structure in water at 4 °C. The resulting 3D structure, composed of a PNIPAM hydrogel, reversibly changes the volume in response to temperature change as shown in Figure 1e.

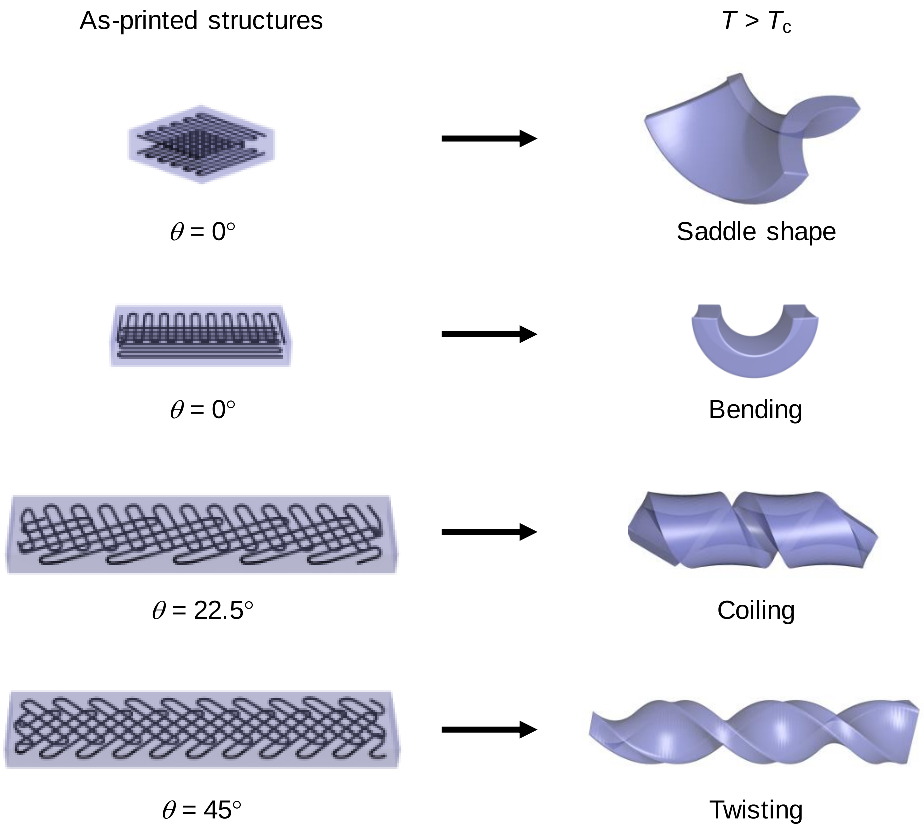

Figure S2. Schematic illustration of programming various motions into 3D structures using bilayer structures that consist of orthogonally oriented linear contractile elements (orthogonally growing bilayer structures). The structures on the left side represent as-printed structures, in which the dark blue lines are PEG reinforcement patterns in PNIPAM hydrgels (light blue). The structures on the right side represent programmed structures at the shrunk state ($T > T_c$). An orthotropically growing bilayer structure with a square shape forms a saddle-like shape at the shrunk state. This saddle-like shape change can be further exploited to produce various motions by controlling the geometry and orientation of the elements. Increasing the aspect ratio of the bilayer structure induces a pure bending-like motion. Controlling the angle θ between the long axis of the structure and the direction of intrinsic curvature produces bending, coiling, and twisting motions with θ of 0°, 22.5°, and 45°, respectively.

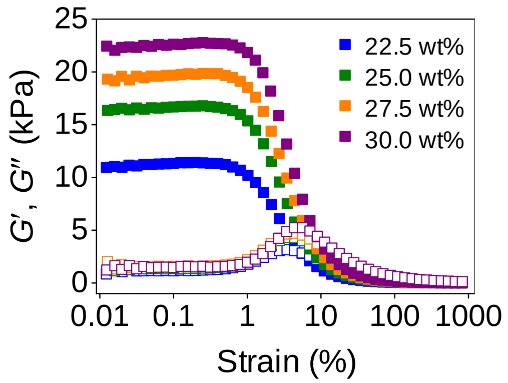

Figure S3. G′ and G″ of the fugitive carriers (pure Pluronic F127 inks) with different concentrations (22.5–30 wt% as shown in the legend) on strain sweeps (0.01%–1000%) at a frequency of 1 Hz, showing shear-thinning properties.

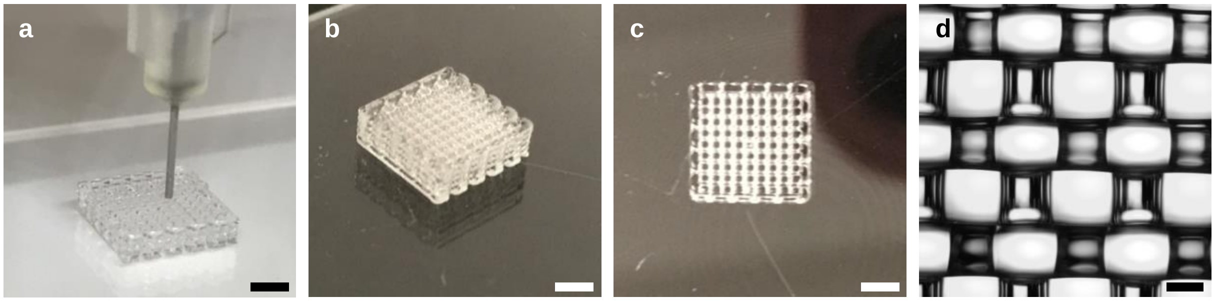

Figure S4. 3D printability of the fugitive carrier ink (25 wt% pure Pluronic F127). a) 3D printing process. b) As-printed lattice structure of the fugitive carrier. c) Top view of the structure. d) Optical microscope image of the structure. Scale bars, 2 mm (a–c); 200 μm (d).

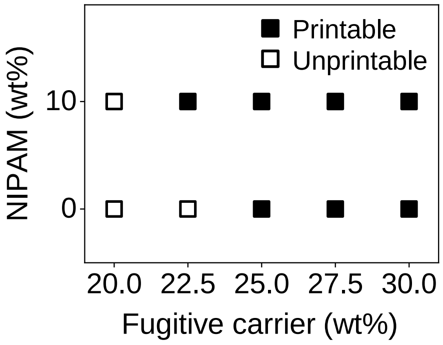

Figure S5. 3D printability of PNIPAM inks (10 wt% NIPAM and 1 wt% PEGDA) with the fugitive carrier (20–30 wt%) and pure fugitive carrier inks (20–30 wt%). The ink (10 wt% NIPAM and 1wt% PEGDA) with the fugitive carrier (22.5 wt% slightly above CMC) is 3D printable, whereas the pure Pluronic F127 ink (22.5 wt%) is not 3D printable. The pure Pluronic F127 ink (22.5 wt%) forms droplets at the nozzle during extrusion, resulting in discontinuous, nonuniform filaments.

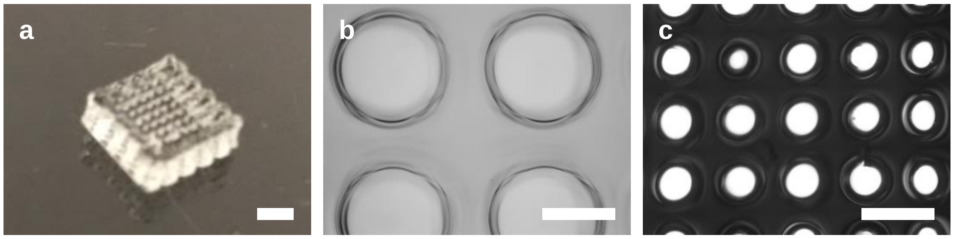

Figure S6. 3D printing using a PNIPAM ink (10 wt% NIPAM and 1 wt% PEGDA) with the fugitive carrier (20 wt%). The concentration of the fugitive carrier in the ink (20 wt%) is lower than its critical micelle concentration (≈21 wt%). a) Optical microscope image of an as-printed 3D lattice structure. The structure shows deformation in the middle (i.e., not self supporting). Although it shows a shear-thinning behavior (Figure 1b), the PNIPAM ink is not 3D printable (based on our definition of 3D printability in this study). b) Optical microscope images (top view) of the structure at the swelled state (25 °C). c) Optical microscope image (top view) of the structure at the shrunk state (40 °C). The round shape of the lattice structure indicates that the ink flows after extrusion and forms the meniscus before crosslinking. Scale bars, 2 mm (a); 500 μm (b, c).

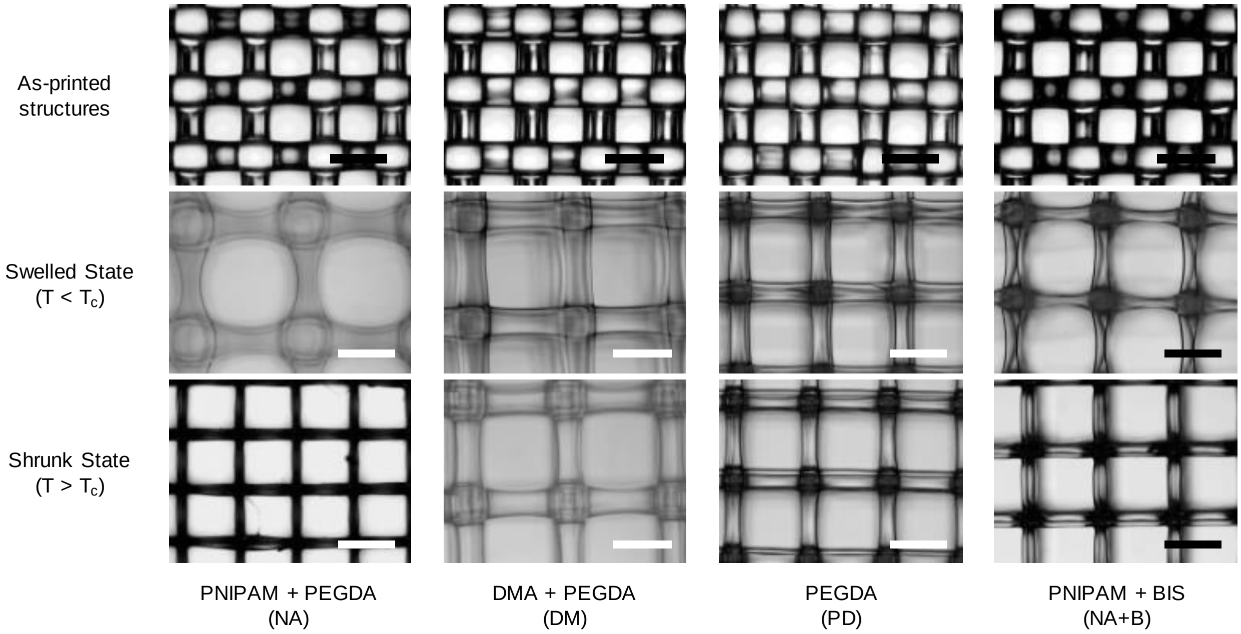

Figure S7. Optical microscope images (top view) of multilayer lattice structures printed with the inks shown in Figure 1j, k. Scale bars, 500 μm.

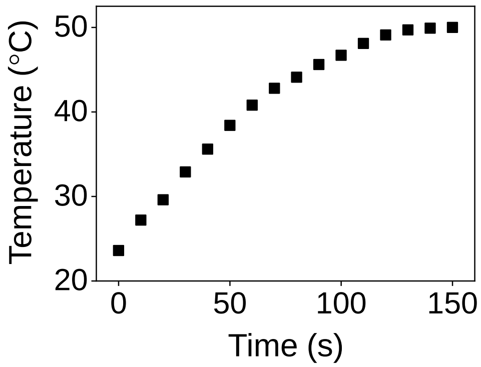

Figure S8. Temperature of a solution used to measure the rate of actuation (Figure 2e) as a function of time. The temperature reaches 35.6 °C at 40 s.

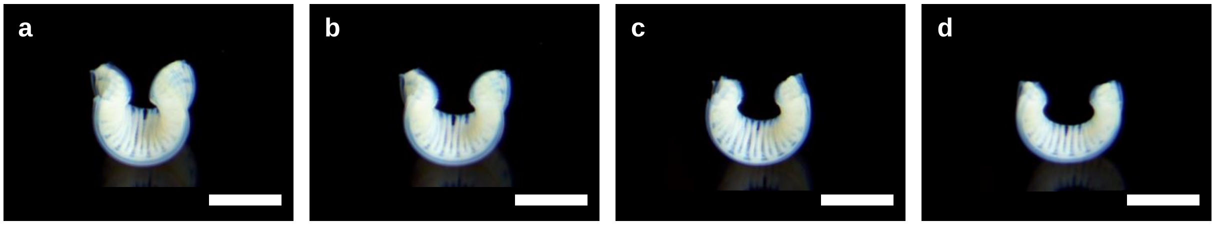

Figure S9. Orthogonally growing bilayer structures with the direction of a principal curvature parallel to the long axis ($\theta = 0°$) with different aspect ratios at the shrunk state. The structures have an as-printed length and thickness of 12 mm and 1.6 mm, respectively, and width of 7.8 mm (a), 6.6 mm (b), 5.4 mm (c), and 4.2 mm (d). Scale bars, 5 mm.

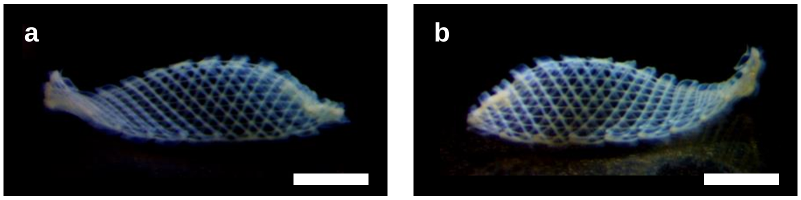

Figure S10. Twisting configurations with θ of 45° (a) and 135° (b) at the shrunk state, showing reversed handedness. Scale bars, 5 mm.

Movie S1. 3D structure showing a bending motion. The movie is shown 100× faster than real time.

Movie S2. 3D structure showing a twisting motion. The movie is shown 100× faster than real time.

Movie S3. Multimodular 3D structure with hybrid motions. The structure consists of 7 modular components: a stationary component, functional components programmed for bending (downward), bending (upward), bending (downward), and linear contraction motions (from left to right), and two wings with twisting motions. The movie is shown 200× faster than real time.

Movie S4. Multimodular 3D structure with hybrid motions. The structure consists of 7 modular components: a stationary component, functional components programmed for bending (downward), bending (upward), linear contraction, and bending (upward) motions (from left to right), and two wings with twisting motions. The movie is shown 200× faster than real time.

Movie S5. Multimodular 3D structure with hybrid motions. The structure consists of 5 modular components: functional components programmed for linear contraction, bending (upward), bending (downward), and bending (upward) motions and a stationary component (from left to right). The movie is shown 200× faster than real time.
